# ICGE: an R package for detecting relevant clusters and atypical units in gene expression

**DOI:** 10.1186/1471-2105-13-30

**Published:** 2012-02-13

**Authors:** Itziar Irigoien, Basilio Sierra, Concepcion Arenas

**Affiliations:** 1 Department of Computation Science and Artificial Intelligence, University of the Basque Country, Donostia, Spain; 2 Department of Statistics, University of Barcelona, Barcelona, Spain

## Abstract

**Background:**

Gene expression technologies have opened up new ways to diagnose and treat cancer and other diseases. Clustering algorithms are a useful approach with which to analyze genome expression data. They attempt to partition the genes into groups exhibiting similar patterns of variation in expression level. An important problem associated with gene classification is to discern whether the clustering process can find a relevant partition as well as the identification of new genes classes. There are two key aspects to classification: the estimation of the number of clusters, and the decision as to whether a new unit (gene, tumor sample...) belongs to one of these previously identified clusters or to a new group.

**Results:**

ICGE is a user-friendly R package which provides many functions related to this problem: identify the number of clusters using mixed variables, usually found by applied biomedical researchers; detect whether the data have a cluster structure; identify whether a new unit belongs to one of the pre-identified clusters or to a novel group, and classify new units into the corresponding cluster. The functions in the ICGE package are accompanied by help files and easy examples to facilitate its use.

**Conclusions:**

We demonstrate the utility of ICGE by analyzing simulated and real data sets. The results show that ICGE could be very useful to a broad research community.

## Background

There is considerable interest among researches in using cluster methods. For example, a common approach in many biomedical applications is to seek a reliable and precise classification of genes into a number of clusters, which is essential for understanding the bases of complex diseases. For instance, an accurate classification of tumors is essential to successful diagnosis and treatment of cancer. Clustering algorithms attempt to partition the units into groups that have similar properties and it is necessary to identify the value of *k *at which the final partition appears to be the best. There is considerable interest among researches in using cluster methods, which can be generally found in R packages on the Comprehensive R Archive Network (CRAN, http://CRAN.R-project.org). An important problem associated with the classification of units is to assess whether the clustering process finds a relevant partition, and to identify new classes of units. For example, if genes are classified into groups exhibiting similar patterns of gene expression variation, it is necessary to pay attention to two things. First the correct classification in *k *clusters of the genes by an unsupervised method. Usually, when a clustering algorithm is applied to a set of units, although the data do not present a cluster structure, the algorithm returns a partition. It is thus necessary that the index used to establish the "real" number of clusters should also be able to detect the absence of cluster structure. A variety of measures for determining the "real" number of cluster can be found in the literature, see for example [1-8] or [9], which gives an excellent overview. Most of these procedures are useful only for continuous data. Only one, the silhouette method [5], is appropriate for any kind of data (continuous, binary or qualitative). Data of this kind are common in biomedical applications, but the silhouette index cannot detect the absence of a cluster structure. An index that can be applied to any kind of attribute type, the *INCA *index, can be found in [10]. This index can use continuous data without any assumption about their distribution and it also permits detection of data that have no cluster structure. Second, given a new gene, the procedure should establish whether it is sufficiently similar to any of the existing clusters. If not, a new type of expression pattern must be considered. Note that some techniques of classification, similar to discriminant analysis, classify a new unit as necessarily belonging to one of the specified clusters. However, this new unit may not belong to any of the pre-identified clusters, but may rather be a member of an entirely different and unknown cluster. There are few approaches in the literature dealing with the typicality problem [11-15]. All these methods have some restrictions on the type of data (only continuous data following normal distribution) or on the number of groups (only two groups for any kind of data). Recently, Irigoien et al. [10] developed an effective test for determining atypical objects in different types of clustering applications. This test provides an alternative to the other models that impose constraints on the type of data or the number of groups. This test can be used with any kind of data, and has no limitation on the number of groups.

The literature on statistical clustering is large, but it does not appear to contain any computational tool capable of solving all the key aspects of classification: identifying the number of clusters using mixed variables, usually found in applied biomedical research; detecting whether the data have a cluster structure; identifying whether a new unit belongs to one of the pre-identified clusters, and classifying this unit. The ICGE package uses the methodology introduced in [10] and deals with all the aspects commented above.

## Implementation

In this section, the structure of the package and the functions implemented are explained. Examples illustrating the usage of the functions are also included.

The ICGE package was developed for the free statistical R environment (http://www.r-project.org) and runs under the major operating systems. We do not delve here into details of the underlying statistical methodology. However, a review of this methodology can be found in the Methods subsection.

### Main functions

Table 1 summarizes the main functions available in the package. A detailed description of these functions is provided below.

**Table 1:** Main functions on package ICGE

Function name	Description
INCAindex	Calculates the *INCA *index.
INCAnumclu	Calculates the *INCA *index for different partitions.
INCAtest	Performs a typicality test.

Name and small description of the main functions in ICGE.

The main function INCAindex helps to estimate the number of clusters in a dataset.

• **Usage**

INCAindex(d, pert_clus)

• **Arguments**

To call the main function INCAindex(d, pert_clus), two arguments must be specified. As usual, d is a distance matrix or a dist object containing the distances between the *n *units, and pert clus is an *n*-vector that indicates which group each unit belongs to. The default value indicates the presence of only one group in the data. Note that the expected values of pert_clus are greater than or equal to 1 (for instance 1,2,3,4...).

• **Value**

This function returns an object of class incaix, which is a list containing the following components:

well_class, a vector indicating the number of units that are well classified;

Ni_cluster, a vector indicating each cluster size and

Total, percentage of units well classified in the partition defined by pert_clus, i.e., the *INCA *index.

• **Remarks **It admits the associated methods summary and plot. The first simply returns the percentage of well-classified units and the second offers a barchart with the percentages of well classified units for each group in the given partition.

• **Example 1**

Consider the following simulated data. Using the data simulation functions included in the WGCNA package (see [16]), we generated 100 samples and three groups containing 480, 360 and 360 genes, respectively. We used the Euclidean distance and calculated the percentage of well classified genes.

library("WGCNA")

library("ICGE")

nSamples = 100

set.seed(3)

nModules = 3

nGenes = 1200

eigengenes = matrix(rnorm(nSamples *nModules), nSamples, nModules)

d = simulateDatExpr(eigengenes, nGenes, c(b0.3, 0.3, 0.4, 0), signed = TRUE)

data = d$datExpr;

dst = dist(t(data))

x = INCAindex(dst, d$allLabels)

The output was: a vector indicating the number of units in each group (360, 360 and 480); the number of units well classified (84, 94 and 91 per cent, respectively) and the *INCA *index, which indicat the total of units well classified (89.84%).

Furthermore, in order to obtain an estimation of the "real" number of clusters from the data, we compute the *INCA *index for several partitions, with different number *k *of clusters in each partition, where *k *= 2,..., K. This is the aim of INCAnumclu function.

• **Usage**

INCAnumclu(d, K, method="pam", pert, noise="NULL", L)

• **Arguments**

The function INCAnumclu(d, K, method="pam", pert, noise="NULL", L) has 6 arguments but they are not involved simultaneously. A distance matrix d or a dist object with distance information between the *n *units is required. Argument K indicates the maximum number of clusters to be considered. For each value *k, k *= 2, ..., *K *a partition in *k *clusters is considered. The method argument is a character string defining the clustering method to be applied in order to obtain the corresponding partitions. The clustering method is performed via the function pam and agnes in cluster package. The available clustering methods are pam (default method, Partitioning Around Medoids clustering method, PAM, [17]), average (UPGMA), single (single linkage), complete (complete linkage), ward (Ward's method), weighted (weighted average linkage). Nevertheless, the user can introduce particular partitions indicating method="partition" and using the pert argument. This argument is a matrix with *n *rows, and each column contains a particular partition. This means that each column is an n vector that indicates the group to which each unit belongs. Note that the expected values of each column of pert are consecutive integers that are greater than or equal to 1 (for instance 1,2,3,4..., k). The argument noise is a logical vector indicating units considered as "noise" units by the user, and argument L must be set as L="custom". When argument L = "NULL" no "noise" units are considered. If parameter L is greater than or equal to 1, the units classified in all clusters *C*, containing a number of units ≤ L, are considered "noise" units. If L="custom", the "noise" units are selected by the user and they must be indicated in the noise argument.

• **Value**

This function returns a numeric vector with the *INCA *index values calculate for each partition (with and without the units considered "noise" units). The associate method plot returns two plots of *INCA*_*k *_*versus *the number of clusters *k*. One plot shows the *INCA*_*k *_index values considering all the units, and the other shows the *INCA*_*k *_index values calculated without "noise" units. As explained in [10], when the value *INCA*_*k*+1 _shows a large decrease respect to the *INCA*_*k *_value, we conclude that there are *k *clusters in the data. When values of *INCA*_*k *_are low and constant, it means that there is no cluster structure, or that all the data form a single cluster.

• **Example 1 (cont.)**

The average clustering algorithm was applied to the same data. Using INCAnumclu we determined the number of clusters. Consequently we calculated the *INCA *index associated with partitions having *k *= 2, ..., 10 clusters.

out <- INCAnumclu(dst,10,"average")

plot(out)

**Figure 1:**
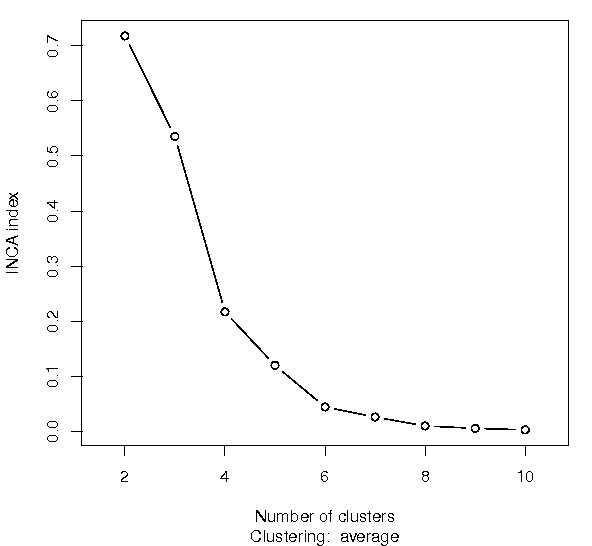
**Estimating the number of clusters using data in example 1 (Implementation section)**. Plot of the index *INCA*_*k *_*versus *the number of clusters *k*. The largest (negative) slope indicates that there are three clusters.

The procedure gives good results and identifies the three clusters, see Figure 1.

• **Example 2**

Now consider the following example. Using the data simulation functions included in [16], we generated 100 samples and three groups containing 360, 360 and 360 simulated genes, respectively. A fourth group with 120 "noise" genes was also generated. The INCAnumclu function shows (taken K = 15) that the "noise" genes have hidden the underlying cluster structure. Using the parameter noise with L = 2, the procedure identifies, in the initial partitions 13 "noise" genes.

library("WGCNA")

library("ICGE")

nSamples = 100

set.seed(3)

nModules = 3

nGenes = 1200

eigengenes = matrix(rnorm(nSamples *nModules), nSamples, nModules)

d = simulateDatExpr(eigengenes, nGenes, c(0.3, 0.3, 0.3, 0.1), signed = TRUE)

data = d$datExpr;

dst = dist(t(data))

out<-INCAnumclu(dst, 15, "average", L = 2)

The results are:

INCA index to estimate the number of clusters considering all units

Clustering method: average

k = 2, 0.00042

k = 3, 0.33

k = 4, 0.25

k = 5, 0.2

k = 6, 0.17

k = 7, 0.14

k = 8, 0.12

k = 9, 0.52

k = 10, 0.54

k = 11, 0.5

k = 12, 0.41

k = 13, 0.41

k = 14, 0.36

k = 15, 0.3

Noise units:

Gene.1081 Gene.1087 Gene.1102 Gene.1107 Gene.1128 Gene.1134 Gene.1141

Gene.1155 Gene.1158 Gene.1165 Gene.1170 Gene.1176 Gene.1182

INCA index to estimate the number of clusters without the noise units

Clustering method: average

k = 2, 0.67

k = 3, 0.44

k = 4, 0.2

k = 5, 0.069

k = 6, 0.0024

k = 7, 0.0022

k = .8, 0.043

k = 9, 0.00087

k = 10, 0.034

k = 11, 0.031

k = 12, 0.028

k = 13, 0.026

k = 14, 0.024

k = 15, 0.023

Finally, INCAtest function performs the typicality *INCA *test. Therein, the null hypothesis that a new unit g_0 _is a typical unit with respect to a previously fixed partition is tested *versus *the alternative hypothesis that the unit is atypical.

• **Usage**

INCAtest(d, pert, d test, np = 1000, alpha = 0.05, P = 1)

• **Arguments**

By calling the function, INCAtest(d, pert, d_test, np = 1000, alpha = 0.05, P = 1), 6 arguments are specified. As before, d is a distance matrix or a dist object with distance information between the *n *units, and pert is an *n*-vector that indicates the group to which each unit belongs. The default value indicates the presence of only one group in the data. Note that the expected values of pert are greater than or equal to 1 (for instance 1,2,3,4...). The argument d_test is a vector of length *n *that contains the distances from the new unit g_0 _to the rest of the *n *units. Note that sampling distributions of the *INCA *statistics *W*(g_0_) and the related statistics *U*_*j*_(g_0_) (*j *= 1, ..., *k*) (see subsection Methods for the definition) can be difficult to find for mixed data, but they may nevertheless be obtained by re-sampling methods, in particular by drawing bootstrap samples as follows. Draw *N *units g with replacement from the union of *C*_1_, ..., *C*_*k *_and calculate the corresponding *W*(g) and *U*_*j*_(g) (*j *= 1, ..., *k*) values. This process is repeated 10P times. In this way, the bootstrap distributions under *H*_0 _are obtained. Then, the np and alpha arguments indicate the sample size for the bootstrap procedure, and the level for the test, respectively. Finally, the argument P indicates that the bootstrap procedure is repeated 10P times.

• **Value**

The function returns a list with incat class containing the following components:

StatisticW0 value of the *INCA *statistic;

ProjectionsU values of statistics measuring the projection from the specific object to each group;

Percentage under alpha percentage of times that the *INCA *test has been rejected for a fixed significance level;

alpha specified value of the significance level of the test.

• **Example 2 (cont.)**

Consider the above simulated gene-expression data that include 120 "noise" genes, 100 samples and three groups containing 360, 360 and 360 simulated genes, respectively.

Now, consider only the three groups without "noise" genes. Select one "noise" gene at random and insert the distances from it to the "non-noise" genes in vector dd. Then, compute the INCAtest function:

dr<-as.matrix(dst)[d$allLabels! = 0,d$allLabels! = 0]

cl<-d$allLabels[d$allLabels! = 0]

INCAtest(dr,cl,dd,np = 1000,alpha = 0.05, P = 1)

As we expected, the output indicates that this "noise" gene is atypical.

StatisticW0

238758.6

ProjectionsU

1 147.4769

2 257.0330

3 433.4185

Percentage under alpha

100

alpha

0.05

Also take at random (from group 3) one gene of the cluster (i.e., "non-noise") genes and insert the distances from it to the "non-noise" genes in vector dd. Then, the INCAtest correctly predicts their cluster membership:

INCA test

INCA statistic value = 5.839681

U projections values:

*U*_1 _= 137.9698

*U*_2 _= 137.3148

*U*_3 _= 7.615953

significative tests for alpha= 0.05: 0

We also considered 100 genes selected at random: 87 "non-noise" genes (27 from group 1, 30 from group 2 and 30 from group 3) and 13 "noise" genes. We computed the INCAtestfunction. The results show that the function correctly predicts the cluster membership of the 87 "non-noise" genes. For the "noise" genes, 8 are considered as atypical and the other 5 are confounded as genes of the initial groups.

### Auxiliary functions

These main functions are, of course, based on the auxiliary functions that calculate the geometric variability, the distance between two groups, the proximity function and the *INCA *statistic itself, which are described at the beginning of the Method Section. Table 2 shows the corresponding functions available from the package, and more detailed comments are presented below.

**Table 2:** Auxiliary functions on package ICGE

Function name	Description
vgeo	Calculates the geometrical variability.
deltas	Calculates the distance between each pair of groups.
proxi	Calculates the proximity function.
estW	Calculates the *INCA *statistic.

Name and small description of the auxiliary functions in ICGE.

The vgeo function calculates the geometrical variability ${\hat{V}}_{\delta }\left(C_{j}\right)$ (see subsection Methods for the definition) for each group in the data.

• **Usage**

vgeo(d, pert = "onegroup")

• **Arguments**

To call vgeo(d, pert = "onegroup") two arguments must be specified. The d argument is a distance matrix or a dist object with distance information between the *n *units and pert is an *n-*vector that indicates the group to which each unit belongs. The default value indicates that there is only one group in the data. Note that the expected values of pert are numbers greater than or equal to 1 (for instance 1,2,3,4...).

• **Value**

The function returns a matrix containing the geometric variability for each group.

The deltas function calculates the distance ${\hat{\Delta }}_{ij}^{2}$ between each pair of groups *C*_*i *_and *C*_*j *_in the data (see subsection Methods for the definition).

• **Usage**

deltas(d, pert = "onegroup")

• **Arguments**

To call deltas(d, pert = "onegroup") the same d and pert arguments must be specified.

• **Value**

The function returns a matrix containing the distances between each pair of groups. proxi function calculates the proximity function ${\hat{\phi }}^{2}\left({\text{g}}_{0},C_{j}\right)$ (see subsection Methods for the definition) from a specific unit g_0 _to the other groups *C*_*j *_in the data.

• **Usage**

proxi(d, dx0, pert = "onegroup")

• **Arguments**

To call proxi(d, dx0, pert = "onegroup") three arguments must be specified. The d argument is a distance matrix or an object dist for the *n *units; dx0 is an *n-*vector containing the distances from g0 to the rest of the units and pert is an *n-*vector that indicates the unit to which group belongs. The default value indicates that there is only one group in the data. Note that the expected values of pert are numbers greater than or equal to 1 (for instance 1,2,3,4...).

• **Value**

The function returns a vector containing the proximity function from g_0 _to each group.

The function estW calculates the *INCA *statistic *W*(g_0_) and the related statistics *U*_*j*_(g_0_), *j *= 1, ..., *k.*

• **Usage**

estW(d, dx0, pert = "onegroup")

• **Arguments**

This needs the same arguments as proxi.

• **Value**

The function returns an object of incaest class, which is a list containing the following components:

Wvalue, is the *INCA *statistic *W*(g_0_);

Uvalue, is a vector containing the statistics *U*_*j*_(g_0_), *j *= 1,..., *k*.

The associated summary method returns only the *INCA *statistic value.

### Distance functions

Note that all these functions require the previous calculation of a distance between units. Biomedical and genetic studies incorporate any type of data, not only continuous variables, and correlation or other types of dissimilarities are frequently used for clustering. For this reason, ICGE can calculate different distance matrices (Table 3).

**Table 3:** Distances provided by the ICGE package

Function name	Description
dbhatta	Bhattacharyya distance
dcor	Correlation distance
dgower	Gower distance with and without missing values
dmahal	Mahalanobis distance
dproc2	Procrustes distance

Name and small description of the distances available in ICGE.

The correlation distance and the Mahalanobis distance [18] are well known, but perhaps the Bhattacharyya and the Gower distances are less. A function named mahalanobis() that calculates the Mahalanobis distance already exists in the stats package, but it is not suitable in our context. While this function calculates the Mahalanobis distance with respect to a given center, our function is designed to calculate the Mahalanobis distance between each pair of units given a data matrix.

The Bhattacharyya distance [19] between two units with frequencies *i *= (*p*_*i*1_, ..., *p*_*i*m_) and *j *= (*p*_*j*1_, ...,*p*_*jm*_) is defined by:

$$d\left(i,j\right)=\text{arccos}\overset{m}{\underset{l=1}{\sum }}\sqrt{p_{il}p_{jl}}.$$

The Gower distance [20], used for mixed variables, is defined by $d_{ij}^{2}=2\left(1-s_{ij}\right)$. As each unit is characterized by *m*_1 _continuous, *m*_2 _binary and *m*_3 _qualitative variables, the similarity coefficient *s*_*ij *_between unit *i *and *j *is calculated as follows:

(1) $$s_{ij}=\frac{\sum_{l=1}^{m_{1}}\left(1-\frac{\left|x_{il}-x_{jl}\right|}{R_{l}}\right)+a+\alpha }{m_{1}+\left(m_{2}-d\right)+m_{3}}$$

where *R*_*l *_is the range of the *l*th continuous variable (*l *= 1,..., *m*_1_); for the *m*_2 _binary variables, *a *and *d *represent the number of matches presence-presence and absence-absence, respectively and *α *is the number of matches between states for the *m*_3 _qualitative variables. Note that there is also the daisy() function in cluster package, which can calculate the Gower distance for mixed variables. The difference between this function and dgower() in ICGE is that in daisy() the distance is calculated as *d*_*ij *_= 1 *- s*_*ij *_and in dgower() as $d_{ij}^{2}=2*\left(1-s_{ij}\right)$. Moreover, dgower() allows us to include missing values (such as NA) and therefore calculates distances based on Gower's weighted similarity coefficients.

The procrustes distance was defined in [21], and it was introduced to find an appropiate distance between genes using their expression profile. It was defined as the procrustes statistics between the procrustes weighted mean associated with two genes, see definition 2, Step C in [21] for more details.

### Methods

Briefly, we describe some of the concepts used in the ICGE package. A more detailed description of the procedure and applications can be found in [10].

Consider a dataset with *n *units and a partition into *k *groups *C*_1_, ..., *C*_*k*_. Let *δ*(g,g') be a distance between units g and g'. Let samples ${\text{g}}_{1}^{1},\ldots ,{\text{g}}_{n_{1}}^{1},\ldots ,{\text{g}}_{1}^{k},\ldots ,{\text{g}}_{n_{k}}^{k}$, of sizes *n*_1_, ..., *n*_*k *_be taken from the *C*_1_, ..., *C*_*k *_groups respectively.

The geometric variability for each group is defined by:

$${\hat{V}}_{\delta }\left(C_{j}\right)=\frac{1}{2n_{j}^{2}}\underset{l,m}{\sum }\delta^{2}\left({\text{g}}_{l}^{j},{\text{g}}_{m}^{j}\right).$$

Given two groups *C*_*i*_, *C*_*j *_the distance between them is given by:

$${\hat{\Delta }}_{ij}^{2}=\frac{1}{n_{i}n_{j}}\underset{l,m}{\sum }\delta^{2}\left({\text{g}}_{l}^{i},{\text{g}}_{m}^{j}\right)-{\hat{V}}_{\delta }\left(C_{i}\right)-{\hat{V}}_{\delta }\left(C_{j}\right).$$

Given the distances from one specific unit g_0 _to the rest of the units organized in the *k *groups, the proximity function of unit g_0 _to *C*_*j *_is defined by:

$${\hat{\phi }}^{2}\left({\text{g}}_{0},C_{j}\right)=\frac{1}{n_{j}}\underset{l}{\sum }\delta^{2}\left({\text{g}}_{0},{\text{g}}_{l}^{j}\right)-{\hat{V}}_{\delta }\left(C_{j}\right).$$

For more details on these concepts see [22].

**Figure 2:**
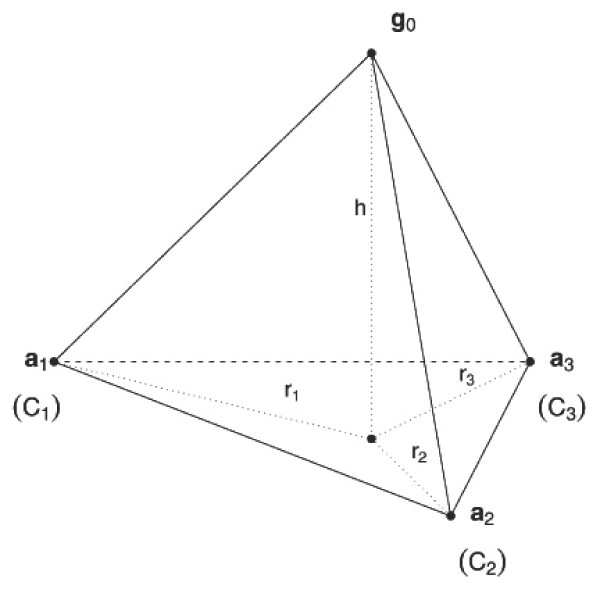
**Geometrical interpretation of the *INCA *statistics**. For *k *= 3, new observation {g_0_}, centers of clusters {a_1_, a_2_, a_3_} and (squared) projection *r*_*i *_of the edges {g_0_, a_*i*_} on the plane {a_1_, a_2_, a_3_}. The (squared) height *h *is the *INCA *statistic.

From these previous concepts we define the *INCA *statistic. Consider a fixed unit g_0_, which may be an element of some *C*_*j*_, *j *= 1,..., *k *or may belong to some unknown cluster, i.e., it may be an atypical unit. This statistic trades off between minimizing the weighted sum of proximities of g_0 _to clusters (which takes into consideration the within-cluster variabilities) and maximizing the weighted sum of the squared distances between clusters (between-cluster variability) - a common feature of a clustering criterion. Moreover, this statistic can be interpreted (see Figure 2) as the (squared) orthogonal distance or height *h *of g_0_ on the hyperplane generated by the centers of *C*_*i *_(*i *= 1,..., *k*), denoted in Figure 2 by a_*i*_, *i *= 1,..., *k*. Then, points which lie significantly far from this hyperplane are held to be atypical. This intuitive idea is used both to determine the number of clusters, and to detect atypical units among existing clusters. The definition of the *INCA *statistic is:

$$W\left({\text{g}}_{0}\right)=\underset{\alpha_{i}}{\text{min}}\left\{L\left({\text{g}}_{0}\right)\right\},\overset{k}{\underset{i=1}{\sum }}\alpha_{i}=1$$

where,

$$L\left(g_{0}\right)=\overset{k}{\underset{i=1}{\sum }}\alpha_{i}\phi_{i}^{2}\left({\text{g}}_{0}\right)-\underset{1\leq i<j\leq k}{\sum }\alpha_{i}\alpha_{j}\Delta_{ij}^{2}.$$

### Estimating the number of clusters

We define the *INCA *index, *INCA*_*k*_, associated with the partition *C*_1_,..., *C*_*k*_, as the probability of finding well classified units. Consider that *n *units are divided into *k *clusters *C*_1_,...,*C*_*k *_of sizes *n*_1_,..., *n*_*k*_, respectively. Fix cluster *C*_*j *_and for each unit g belonging to the data set, consider the value of *INCA *statistic, $W_{C_{j}}\left(\text{g}\right)$, with respect to clusters *C*_*i *_with *i *≠ *j*. Consider the maximum, $W_{C_{j}}$, of these (squared) orthogonal distances for all the units that do not belong to *C*_*j*_. Then consider the following criterion: Unit g of *C*_*j *_is well classified in *C*_*j *_if $W_{C_{j}}\left(\text{g}\right)>W_{C_{j}}$. Unit g of *C*_*j *_is poorly classified in *C*_*j *_if $W_{C_{j}}\left(\text{g}\right)\leq W_{C_{j}}$, in fact, it is closer to another cluster.

Let *N*_*j *_be the total number of units in *C*_*j *_which are well classified. Thus we define the *INCA *index, *INCA*_*k*_, associated with the partition *C*_1_,..., *C*_*k *_as the frequency of well classified, i.e.,

$$INCA_{k}=\frac{1}{k}\overset{k}{\underset{j=1}{\sum }}\frac{N_{j}}{n_{j}}.$$

### Procedure for detecting an atypical observation

Suppose now that a cluster analysis is performed and the optimal number of clusters is found. Let g_0 _be a new unit and consider the *INCA *test to decide whether g_0 _belongs to one of the fixed clusters *C*_*j*_, *j *= 1,..., *k *or, on the contrary, whether it is an atypical observation, belonging to some different and unknown cluster. Compute *W*(g_0_): if this value is significant it means that g_0 _comes from a different and unknown cluster. Otherwise, we allocate g_0 _to *C*_*i *_using the rule:

(2) $$\text{allocate}\;{\text{g}}_{0}\;\text{to}\;C_{i}\;\text{if}\;U_{i}\left({\text{g}}_{0}\right)=\underset{j=1,\ldots ,k}{\text{min}}\left\{U_{j}\left({\text{g}}_{0}\right)\right\},$$

where $U_{j}\left({\text{g}}_{0}\right)=\phi_{j}^{2}\left({\text{g}}_{0}\right)-W\left({\text{g}}_{0}\right)$, *j *= 1, ..., *k*.

For a geometric interpretation, see Figure 2, where for simplicity the (squared) projection *U*_*j*_(g_0_) is denoted by *r*_*j*_, *j *= 1,..., *k*. So, the above criterion follows the next geometric and intuitive allocation rule:

(3) $$\text{allocate }{\text{g}}_{0}\;\text{to}\;\;C_{i}\;\text{if the projection }U_{i}\left({\text{g}}_{0}\right)\text{ is the smaller}\text{.}$$

A more detailed explanation of these procedures, properties and examples can be found in [10].

## Results

### Remarks and limitations of the package

For ICGE package the computing times are reasonable. Table 4 shows runtime for functions INCAindex and INCAtest based on synthetic data sets of different sample sizes (*n *= 50,100, 500 or 1000), and different number of groups *k *= 2, 3, 5, 10, 15, 20, 30 or 40. Observe that for a large number of clusters, the time increases exponentially.

**Table 4:** Runtime for functions INCAindex and INCAtest based on synthetic data sets of different sizes

Function	*n*	*k *= 2	*k *= 3	*k*= 5	*k *= 10	*k *= 15	*k *= 20	*k *= 30	*k *= 40
INCAindex	50	0.046"	0.09"	0.114"	0.527"	1.567"	3.46"	11.018"	25.131"
	100	0.012"	0.0.089"	0.22"	1.059"	3.066"	6.784"	21.291"	49.394"
	500	0.155"	0.535"	1.318"	5.962"	16.555"	35.726"	110.015"	252.279"
	1000	0.37"	1.319"	3.122"	13.317"	35.965"	76.437"	230.809"	522.797"
INCAtest	50	5.446"	6.817"	10.685"	26.341"	51.138"	84.722"	176.928"	304.172"
np = 1000	100	7.718"	9.158"	13.208"	29.651"	54.897"	90.035"	185.46"	318.486"
	500	168.429"	167.676"	169.206"	190.446"	207.657"	284.543"	428.009"	641.595"
	1000	600"	581.966"	571.32"	615.7"	626.671"	697.621"	848.343"	1091.247"

Runtime for functions INCAindex and INCAtest using synthetic data sets with different sample sizes, *n *= 50,100,500 or 500, and different number of groups *k *= 2,3,5,10,15,20,30 or 40. Function INCAtest is performed for np = 1000 resampling units.

Furthermore, note that in the main functions INCAindex, INCAnumclu and INCAtest the argument d is a distance matrix or a dist object. Therefore, any kind of dissimilarity can be used, not only those included in ICGE, and in this sense the package is flexible.

Another aspect is also relevant. Let *p *be the dimensionality of the Euclidean space in which the original metric space (*S, δ*) can be embedded (see [10], section 2). If the number of clusters *k *is equal to or greater than the dimensionality *p*, the hyperplane generated by the cluster centers will simply be the whole space, and the *INCA *statistic will always be zero. This special situation should be taken into account when using these functions, and it may be a limitation of the method (and of the package).

### Application to real and simulated data

Functions and methods are illustrated and tested on both real and simulated data.

#### Chowdary's data set

**Figure 3:**
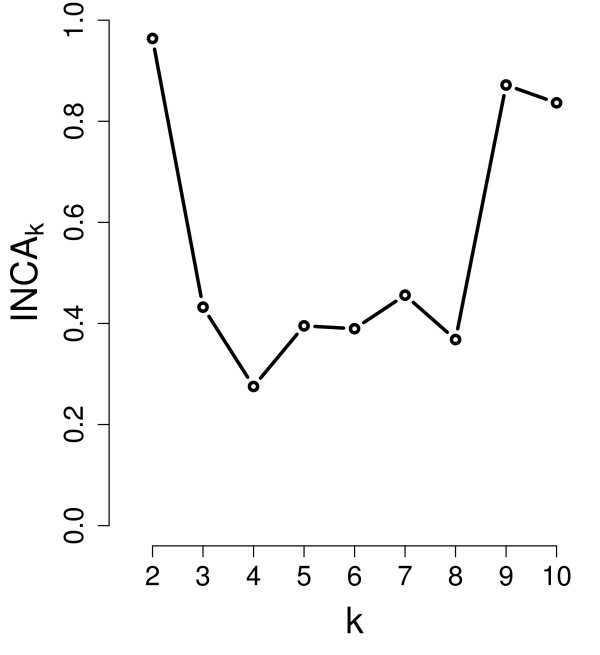
**Estimating the number of clusters using Chowdary's data set**. Plot of the index *INCA*_*k *_*versus *the number of clusters *k*. The largest (negative) slope indicates that there are two clusters.

Chowdary et al., compared in [23] pairs of snap-frozen and RNAlater preservative-suspended tissue from lymph node-negative breast tumors (B) and Dukes' B colon tumors (C). ICGE package proved to be effective at automatically discovering the both groups (see Figure 3). The procedure chooses *k *as the value of *k *prior to the first biggest slope decrease. Using the correlation distance, the clustering procedure PAM was used to partition the 94 samples successively into 2, 3, ..., 10 clusters. The plot indicates that there are two clusters as it was already reported. This data set is included in the package.

#### Golub's data set

Golub et al., studied in [24] gene expression in two types of acute leukemia: acute lymphoblastic leukemia (ALL) and acute myeloid leukemia (AML). They worked with 27 ALL and 11 AML samples. There were no missing values and we standardized the data as described in [25]. We evaluated the performance of the typicality test using the correlation distance. We considered 5 and 3 units at random from ALL and AML group, respectively. Using ICGE we decided whether these unknown units are typical units with respect to ALL and AML groups or whether on the contrary, they are units from another group. We repeated this procedure ten times for each group. Good results at the 5% level were obtained. In each case, the units were identified as units from one of the two groups and were well classified. Data can be found in the multtest library.

#### Synthetic time course data

**Figure 4:**
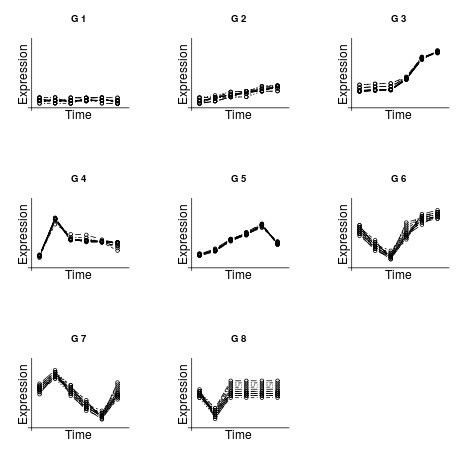
**Synthetic time course data**. Synthetic time course data following 8 different profiles: *G*_1_, constant profile; *G*_2_, monotone increasing but with small difference between the expression value at the first and the last time points; *G*_3_, constant profile at 1, 2 and 3, and later monotone increasing; *G*_4 _up-down profile with maxima at 2; *G*_5_, up-down with maxima at 5; *G*_6_, down-up profile with minima at 3; *G*_7_, cyclic with maxima at 2 and minima at 5; *G*_8_, down-up constant profile with minima at 2 and constant at 3,4,5 and 6.

We generated time course data with 8 groups, 15 genes in each group and six time points, following 8 different profiles (see Figure 4): *G*_1_, constant profile; *G*_2_, monotone increasing but with small difference between the expression value at the first and the last time points; *G*_3_, constant profile at 1, 2 and 3, and later monotone increasing; *G*_4 _up-down profile with maxima at 2; *G*_5_, up-down with maxima at 5; *G*_6_, down-up profile with minima at 3; *G*_7_, cyclic with maxima at 2 and minima at 5; *G*_8_, down-up constant profile with minima at 2 and constant at 3,4,5 and 6. The procrustes distance was used [21]. When genes in *G*_1 _are considered as new genes to be classified in *G*_2_, *G*_3_, *G*_4_, *G*_5_, *G*_6_, *G*_7 _or *G*_8 _the procedure identifies the 15 as belonging to a new group. When genes in *G*_2 _are considered as new genes to be classified in *G*_1_, *G*_3_, *G*_4_, *G*_5_, *G*_6_, *G*_7 _or *G*_8 _the procedure identifies 3 as belonging to a new group (as we know) and 12 as belonging to *G*_1_. When genes in *G*_*i*_, i = 3,4,5,6,7,8 are considered as new genes to be classified in *G*_*j*_, *j *≠ *i*, the procedure identifies the 15 genes as belonging to a new group (as we know). ICGE package proved to be effective at automatically discovering atypical genes. This data set is included in the package.

#### Lymphatic cancer data

**Figure 5:**
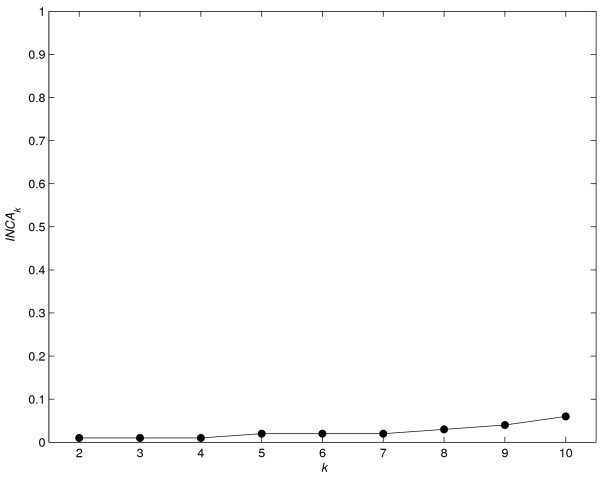
**Lymphatic cancer data**. The low values of the index indicate no cluster structure.

The data from [26] demonstrates that ICGE can correctly identify situations in which the data do not present a clear cluster structure (see Figure 5). The Lymphatic data set consists of 148 instances of the diagnosis of four lymphatic cancer classes (normal found, metastases, malign lymph and fibrosis), with 2, 81, 61 and 4 samples, respectively. Note that it is very difficult for any method to find four clusters given the small sizes of two of the groups. 18 mixed variables were measured: 1 quantitative; 9 binaries and 8 multi-state. ICGE can correctly identify situations in which the data do not present cluster structure. Notice that this is an advantage over other procedures. The PAM clustering procedure was used to partition the 148 samples successively into 2, 3, ..., 10 clusters. Gower's distance was used. As expected, the low values of the index indicate no cluster structure. This data set is included in the package. For additional details read [10].

## Conclusions

ICGE offers a friendly implementation for R users that is capable of solving important questions in genetic analysis and in general studies, where an unsupervised classification is necessary. One aspect of the package is the estimation of the number of clusters. The ICGE procedures provide functionalities that are not offered by other tools; in particular, they can deal with mixtures of categorical and continuous data, a situation usually found by applied researchers. Furthermore, it can detect the absence of cluster structure. Only the silhouette method is appropriate for any kind of data, but this index cannot detect the absence of cluster structure. Thus, our method is able to deal with data of a more general kind. In contrast to other classification techniques, given a new unit to be classified, it does not automatically classify it in previously specified clusters. The procedure decides whether a new unit belongs to a new group. For this reason, the ICGE package is able to solve the typicality problem. Other methods present restrictions in the kind of data or number of groups, but the ICGE package can work with any kind of data and has no limitation on the number of groups. For all these reasons ICGE could be very useful to a large number of researchers.

## Availability and requirements

The ICGE package has been developed for the free statistical R environment (http://www.r-project.org) and will run under the major operating systems. The functions in the ICGE package are accompanied by help files and simple examples to facilitate its use. A manual is also included. ICGE and its documentation are freely available at http://www.sc.ehu.es/ccwrobot.

Software name: ICGE

Software home page: http://www.sc.ehu.es/ccwrobot

Operating system(s): e.g. Platform independent

Programming language: R platform

Other requirements: No

Any restrictions to use: it is available for free download.

## Authors' contributions

II developed the algorithms, prepared the implementation of the ICGE package and carried out the data analysis. BS worked on the draft preparation and cooperated in writing the manuscript. CA conceived the software implementation idea, drafted and wrote the manuscript. II and CA designed the statistical method. All authors have read and approved the final version of the manuscript.
